# Morbillivirus Glycoprotein Expression Induces ER Stress, Alters Ca^2+^ Homeostasis and Results in the Release of Vasostatin

**DOI:** 10.1371/journal.pone.0032803

**Published:** 2012-03-05

**Authors:** Jean-Marc Brunner, Philippe Plattet, Marie-Agnès Doucey, Lia Rosso, Thomas Curie, Alexandra Montagner, Riccardo Wittek, Marc Vandelvelde, Andreas Zurbriggen, Harald Hirling, Béatrice Desvergne

**Affiliations:** 1 Center for Integrative Genomics, University of Lausanne, Lausanne, Switzerland; 2 Institut de Biotechnologie, University of Lausanne, Lausanne, Switzerland; 3 Department of Clinical Research and Veterinary Public Health, Vetsuisse Faculty, University of Bern, Bern, Switzerland; 4 Division of Experimental Oncology, Multidisciplinary Oncology Center, Centre Hospitalier Universitaire Vaudois, University of Lausanne, Lausanne, Switzerland; 5 Brain Mind Institute, Faculté des Sciences de la Vie, Ecole Polytechnique Fédérale de Lausanne EPFL, Lausanne, Switzerland; Virginia Polytechnic Institute and State University, United States of America

## Abstract

Although the pathology of *Morbillivirus* in the central nervous system (CNS) is well described, the molecular basis of neurodegenerative events still remains poorly understood. As a model to explore *Morbillivirus*-mediated CNS dysfunctions, we used canine distemper virus (CDV) that we inoculated into two different cell systems: a monkey cell line (Vero) and rat primary hippocampal neurons. Importantly, the recombinant CDV used in these studies not only efficiently infects both cell types but recapitulates the uncommon, non-cytolytic cell-to-cell spread mediated by virulent CDVs in brain of dogs. Here, we demonstrated that both CDV surface glycoproteins (F and H) markedly accumulated in the endoplasmic reticulum (ER). This accumulation triggered an ER stress, characterized by increased expression of the ER resident chaperon calnexin and the proapoptotic transcription factor CHOP/GADD 153. The expression of calreticulin (CRT), another ER resident chaperon critically involved in the response to misfolded proteins and in Ca^2+^ homeostasis, was also upregulated. Transient expression of recombinant CDV F and H surface glycoproteins in Vero cells and primary hippocampal neurons further confirmed a correlation between their accumulation in the ER, CRT upregulation, ER stress and disruption of ER Ca^2+^ homeostasis. Furthermore, CDV infection induced CRT fragmentation with re-localisation of a CRT amino-terminal fragment, also known as vasostatin, on the surface of infected and neighbouring non-infected cells. Altogether, these results suggest that ER stress, CRT fragmentation and re-localization on the cell surface may contribute to cytotoxic effects and ensuing cell dysfunctions triggered by *Morbillivirus*, a mechanism that might potentially be relevant for other neurotropic viruses.

## Introduction

Canine distemper virus (CDV), closely related to measles virus (MV), belongs to the *Morbillivirus* genus of the *Paramyxoviridae* family. The CDV genome consists of a non-segmented, single-stranded RNA molecule of negative polarity. Both CDV and the human pathogen MV may induce dramatic complications in the central nervous system (CNS). Due to similarities between CDV-mediated demyelination and human multiple sclerosis (MS), the canine disease represents one of the few spontaneously occurring animal models to study the pathogenesis of myelin loss associated with infectious and immune-mediated mechanisms [1]. Epidemiological observations strongly suggest that MS is a disease caused by an infectious agent that induces an immune-mediated demyelinating disease. While much progress has been made in recent years, the pathogenesis of MS is still unclear, and animal models of viral demyelination remain important tools in MS research. Common to most animal models of viral demyelination is viral persistence, the driving force behind the progression of the disease. Thus, understanding the mechanisms of viral persistence might contribute to our understanding of chronic demyelinating diseases. For these reasons, CDV is considered as a model for human multiple sclerosis, as well as for the study of *Morbillivirus*-mediated pathogenesis [1], [2].

We previously found that a recombinant CDV (rgA75/17-V), closely related to the wild-type neurovirulent A75/17 strain, could infect neuron and astrocyte primary cultures from the rat brain, inducing a persistent, non-cytolytic infection. Using this model, we demonstrated that, following CDV infection, glutamate release in the extra-cellular compartment was involved in the induction of neuronal cell death in infected and neighbouring non-infected cells [3].

ER stress is caused by conditions that perturb ER functions including calcium release and accumulation of misfolded protein [4], [5]. When misfolded proteins accumulate in the ER, a stereotypical cellular program called the unfolded protein response is activated, which allows the cell to restore physiological conditions [4], [5]. Initially the cell reacts by expressing more chaperons, such as calreticulin (CRT), but, under persistent stress such as observed in viral infection, the unfolded protein response switches from being pro-survival to proapoptotic [5], [6]. At this stage, the cell starts to transcribe proapoptotic transcription factors such as the growth arrest- and DNA damage-inducible gene 153 (CHOP/GADD 153) [7], [8].

In attempts to characterize the mechanisms of ER stress that might be induced by CDV infection, we focused our attention on the 60-kDa molecular chaperon CRT. This protein has been shown to modulate the homeostasis of calcium (Ca^2+^) in the cell [9]. We demonstrated that in Vero cells and primary hippocampal neurons the CDV surface glycoproteins markedly accumulated in the ER. This was correlated with a strong upregulation of the molecular chaperons CRT and calnexin, two ER stress-dependent proteins. Over-expression of the proapoptotic transcription factor CHOP/GADD 153 was also demonstrated. Importantly, ER stress and CRT over-expression were closely associated with increase in cytosolic Ca^2+^. Finally, in an unanticipated manner, we detected the 27-kDa N-terminal CRT cleavage product, also termed vasostatin, in CDV infected cells. Remarkably, we demonstrated the presence of CRT N-terminal fragments at the cell surface of both infected and neighbouring non-infected cells, an event that may contribute to the CDV and other virus-mediated neurodegeneration.

## Materials and Methods

### Viruses and plasmids

The previously reported recombinant A75/17-V virus contains an additional transcription unit coding for the enhanced green fluorescent protein (e-GFP) in the 3′ proximal position in the genome, generating rgA75/17-V [10]. To simplify the nomenclature we named the recombinant Vero-cells adapted rgA75/17-V as “CDV”.

Construction of expression plasmids: The plasmids pF-CDV, pF-CDV-ER, pH-CDV and pN-CDV were described previously [10], [11]. After expression, the CDV proteins are named F CDV, F-ER CDV, H CDV and N CDV proteins, respectively. The constructs were made in the mammalian expression vector pCI (Promega, Madison, USA) using PCR and recombinant PCR techniques (Pfu Turbo DNA polymerase, Stratagene-Agilent, Santa Clara, USA). All plasmid sequences were confirmed by automated nucleotide sequence analysis.

### Cell culture, infection, and transfection

Vero cells (ATCC, CCL-81) were grown in Dulbecco's modified Eagle's medium (DMEM) (Sigma, St. Louis, USA) supplemented with 10% fetal calf serum (FCS), penicillin, and streptomycin (Gibco, invitrogen group, Carlsbad, USA) and all the cultures were incubated at 37°C in a humidified atmosphere containing 5% CO_2_, as previously described [10]. Hippocampal rat brain cells were prepared from new born rats [12] and were approved (No. 1150.4) by the cantonal regulation of animal care. Hippocampi without dentate gyri were dissociated with papain and triturated using a glass pipette. After centrifugation at 400 *g* for 2 minutes, cells were plated on individual wells (35 mm) of 6-well plates, each containing poly-D-lysin/laminin-coated borosilicate coverslips (15 mm diameter) at a density of 250000 cells/dish in DMEM 10% FCS. The medium was changed after 3 h to a Neurobasal/B27 medium (Invitrogen, Carlsbad, USA).

One day after seeding, Vero cell cultures at 90% of confluence were infected with CDV at the multiplicity of infections (MOI) of 0.03. Hippocampal rat brain cells were infected with CDV two days after seeding at a MOI of 0.003. Transfection were performed one day after seeding using Lipofectamin (Invitrogen, Carlsbad, USA) (1 mg of each plasmid as indicated in the figure legends, in 2 ml Lipofectamine 2000™) for a period of 24 hrs. Transfections were performed in 35 mm dishes. For calcium signal analyses, Vero cells and hippocampal rat brain cells were transfected transiently for a period of 24 hours with Lipofectamin 2000™ (Invitrogen, Carlsbad, USA) (1 µg DNA per 2,5 µL of Lipofectamin 2000™ for Vero cells and 1,8 µg DNA per 3,3 µL of Lipofectamin 2000™ for hippocampal rat brain cells) in a 35-mm dish. Transfection was done for 2 hours at 37°C, 5% CO_2_ and all plasmids were transfected in equal quantities.

### Immunofluorescence staining

The following mouse monoclonal antibodies were used: anti-calreticulin (CRT) C-terminal domain (CRT-C-term) (Becton and Dickinson BD Bioscience, Erembodegem, Belgium), anti-calnexin (Abcam, Cambridge, UK), anti-C/EBP-homologous protein (CHOP/GADD 153) (Santa Cruz Biotechnology, Santa Cruz, USA), anti-CDV nucleoprotein (D110) [13], anti-Flag and anti-HA (Sigma, St. Louis, USA), anti-GAPDH (cell Signalling, Danvers, USA), anti-hrp, (Sigma, St. Louis, USA). Also were used rabbit polyclonal sera against CDV F and H proteins [11], anti-CRT N-terminal domain (CRT-N-term, kindly provided by Dr. Daniel Law University of Geneva [14] ^,^ anti-Flag (Sigma, St. Louis, USA), anti-HA (Sigma, St. Louis, USA),^ anti-^wheat germ agglutinin (^WGA)^ Alexa 405 conjugated (Invitrogen, Carlsbad, USA), and anti-hrp, (Sigma, St. Louis, USA). The secondary antibodies were FITC- (Sigma, St. Louis, USA), CY3- (Chemicon, Temecula, USA), CY5- (Jackson ImmunoResearch Laboratories, Suffolk, UK) or Alexa 594 (Invitrogen, Carlsbad, USA) conjugated antibodies.

For CRT C-terminal immunofluorescence, infected or transfected cell cultures were fixed in 100% methanol for 10 minutes at −20°C. The fixed cultures were washed in a phosphate saline buffer (PBS). Cultures were blocked in a blocking solution (5% normal goat serum in PBS 1%) for 10 minutes, followed by staining with the CRT-C-terminal antibody. For all the other antibodies and antisera, cultures were fixed in 4% paraformaldehyde for 20 min at 4°C. Cells were then permeabilized (0.1% Triton X100 in PBS) for 10 minutes and blocked in a blocking solution (5% normal goat serum in PBS) for 1 hour, followed by staining with the different antibodies. Incubation with the various antibodies and antisera was performed overnight at 4°C. All antibodies were diluted in a blocking solution. The secondary antibody was added for 1 hour at RT. After intensive washing, cell nuclei were stained with 4′6-diamidino-2-phenylindole (DAPI, Sigma, St. Louis, USA) and subsequently examined by Laser Scanning Confocal microscopy. All images were taken with a Zeiss LSM 510 Meta confocal microscope, the Zeiss LSM 510 confocal scan head was coupled with an Axiovert 200 M microscope (Carl Zeiss, Jena, Germany).

### Calcium signal analyses

To follow Ca^2+^ signals, GFP-aequorin (GA) was used. GA is a fusion protein between aequorin and GFP, which emits luminescent signals when it binds 3 Ca^2+^ ions. This requires the presence of coelenterazine for the intracellular regeneration of the photoprotein [15]. When Ca^2+^ binds to the aequorin protein, it undergoes a conformation change that results in the oxidation of coelenterazine and the emission of a single photon. In the jellyfish, binding of Ca^2+^ to aequorin results in an intermolecular non-radiative energy transfer to GFP. This process is known as chemiluminescence resonance energy transfer (CRET). In our case, GFP and aequorin were fused genetically and an intramolecular transfer of Ca^2+^ activated aequorin luminescence energy to GFP occurred, resulting in the emission of a green light [16], [17]. GA is therefore a bi-functional reporter, whereby expression patterns can be monitored by GFP fluorescence, while Ca^2+^ activities can be measured by GFP fluorescence with single-cell resolution.

GA expression was obtained via transfection of the corresponding recombinant plasmid. Coelenterazine (Interchim, Montluçon, France) is membrane permeable and was added to the transfected cells at 5 mM, for pre-incubation at 37°C for 60 min in a tyrode buffer containing Ca^2+^. In Vero cells, Ca^2+^ signals were then recorded in phenol red-free DMEM, L-glutamine (2 mM), FCS 10%, Hepes (20 mM) medium while hippocampal rat brain cells were recorded in a medium containing NaCl (128 mM), KCl (5 mM), CaCl_2_ (2,7 mM), glucose (10 mM), Hepes (20 mM), MgCl_2_ (1 mM), pH 7,4.

The calibration of photon data was carried out as follows. Light emission is expressed as the fractional rate of photoprotein consumption, which is the ratio between the emission of Light (L, minutes) from that time point and the integral of total light emission from that point until full exhaustion of the photoprotein (Lmax). Photons were counted over 60 seconds every 10 minutes during 24 hours, by using a photon counting system (ACTIMETRICS, Wilmette, IL). After 17 hours of recording in Vero cell cultures, or 21 hours for the hippocampal rat brain cells, ionomycin (2 mM) was added to release all Ca^2+^ from ER stores and from the cells. A high CaCl_2_ solution (10 mM) was then added in order to quantify the total amount of photoprotein (Lmax). Each sample was analysed in triplicate in three separate experiments.

### Western Blot

48 hours after infection, Vero cells were washed in PBS at 4°C, then exposed to a lysis buffer (M-PER Mammalian Protein Extraction Reagent, PIERCE-Perbio bioscience, Alost, Belgium) supplemented with protease inhibitors (Halt Protease Inhibitor Cocktail, EDTA-Free, PIERCE-Perbio bioscience, Alost, Belgium). Cell lysates were scraped off culture dishes and centrifuged (14000 g; 10 minutes at 4°C) to recover cytosolic proteins in the supernatant. Proteins were quantified using nanodrop. The samples were diluted in adequate volumes of standard denaturating buffer. Protein (25 µg/lane) were separated by sodium dodecylsulfate-polyacrilamide gel electrophoresis (SDS-PAGE) at 120 V for 1 hour and 30 minutes on 12% acrylamide gels, and subsequently transferred to PVDF membranes (Westran Membrane, Schleicher & Schuell Bioscience, Dassel, Germany). The membranes were then saturated with 5% skim milk in Tris Buffered Saline (TBS) containing 0.1% Tween 20 for 1 hour at RT. They were then incubated with primary antibody diluted at 1/2500 for the anti-CRT C-terminal, 1/500 for the anti–CRT N-terminal domain and 1/1000 for anti-GAPDH in TBS containing 1% skim milk and 0.1% Tween 20. After 3 rinses in PBS/Tween 20, membranes were incubated 1 h at RT with secondary antibody (diluted at 1/10000 for anti-rabbit IgG HRP, and 1/5000 for anti-mouse IgG HRP in the same TBS/Tween 20 skimmed milk solution). After secondary antibody removal, blots were developed using the enhanced chemi-luminescence (ECL) detection system (Amersham, Piscataway, USA). Each sample was analysed in triplicate in three separate experiments.

### Quantification of viral protein and CRT expression by cytofluorimetry

**Figure 1:**
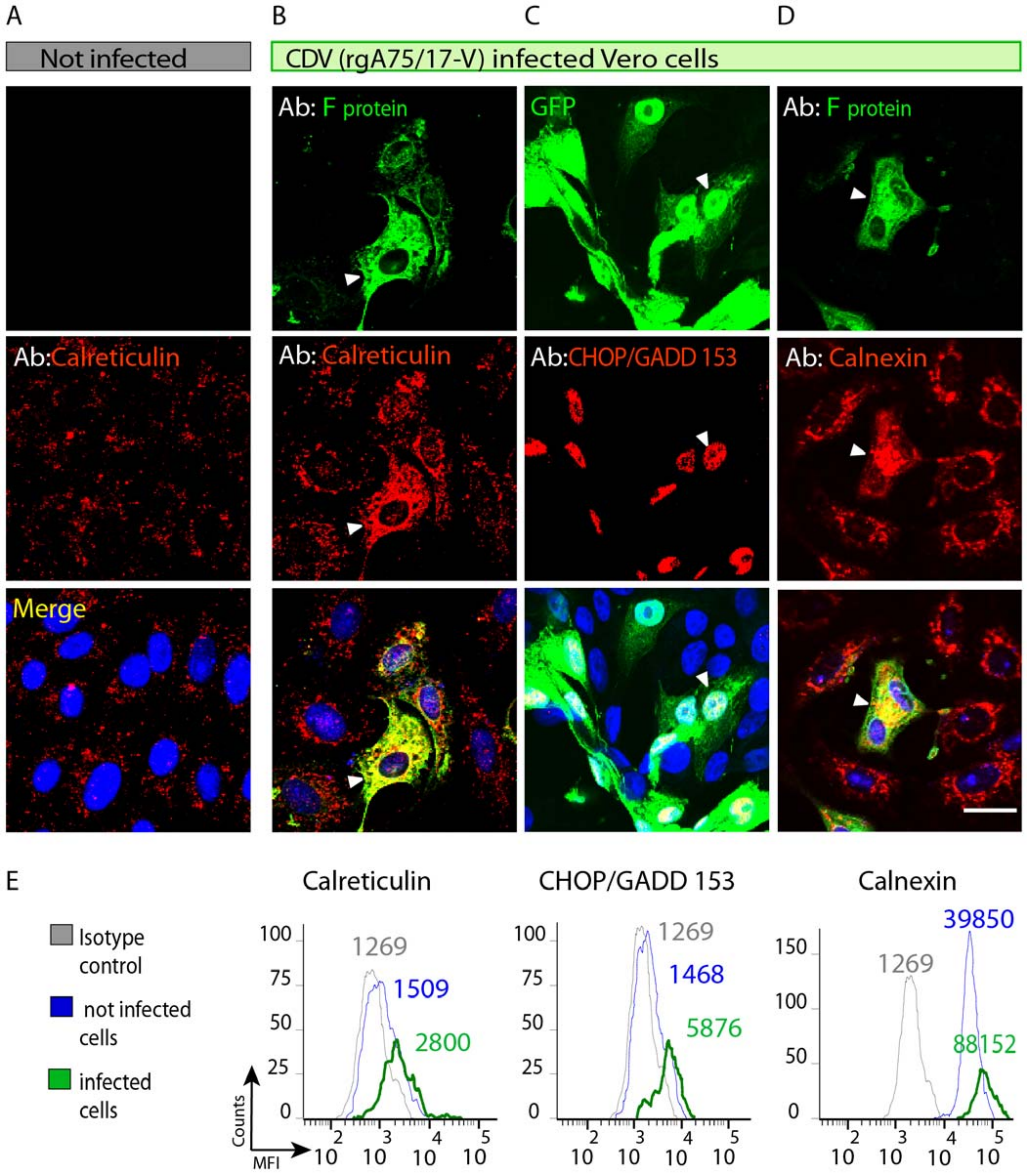
Infection of Vero cells by rgA75/17-V (CDV) induces ER stress. (**A, B, C, D**) Representative photomicrographs of non-infected Vero cells (A) and infected with a Vero cell-adapted canine distemper virus (CDV) strain (rgA75/17-V). The former recombinant CDV expresses the enhanced green fluorescence protein (e-GFP) for easier identification of infected cells (B, C, D). Cultures were infected 1 day after seeding. Cells were then fixed and permeabilized and subsequently analysed by immunofluorescence at 24 hours (A, B and D) or 48 hours (C) after infection. Antibodies against the protein F of CDV (F), calreticulin, CHOP-GADD and Calnexin are as indicated in the panels. Merged images are shown on bottom panels, including labelling with 4′6-diamidino-2-phenylindole (DAPI, blue). Scale bar, 30 µm. Calreticulin and calnexin expression are increased in infected cells that expressed the F protein (B and D) and at 48 hours post-infection, infected cells also express strongly the nuclear proapoptotic CHOP/GADD 153 (C). (**E**) Increase of CRT, CHOP/GADD 153 and calnexin during culture infection, as determined by flow cytofluorimetry. Each sample was analysed in triplicate on three separate experiments, and one representative experiment is shown here.

Twenty four hours post infection with recombinant CDV (rgA75/17-V)-GFP virus, Vero cells were trypsinized and re-seeded in new plates at 5×10^5^ cells. In these series of experiments, to ascertain cell surface staining, cells were mechanically recovered in ice cold PBS containing 2 mM EDTA at 12, 24, 36 and 48 hours, respectively and fixed in a BD FACS™ fixation solution, which preserves plasma membrane integrity (Becton and Dickinson BD Bioscience, Erembodegem, Belgium). Alternatively, in figure 1E, cells were recovered in a similar way 36 hours post infection but treated with the BD FACS™ Perm 2 fixation/permealization solution (Becton and Dickinson BD Bioscience, Erembodegem, Belgium). After blocking with PBS containing 1% goat serum, the cells were stained for one hour at 4°C using antisera or antibodies specific for F protein or CRT N- and C-terminal domains, and secondary goat anti-rat and anti-mouse-Cy5 were added for one hour at 4°C. Cell fluorescence was monitored by flow cytometry using FACSCalibur™ and FACSDiva software (Becton and Dickinson BD Bioscience, Erembodegem, Belgium). One representative experiment was shown of three independent experiments.

## Results

### CDV infection induces ER stress

We previously demonstrated that CDV infection of rat hippocampal primary cultures induced neuronal death in a glutamate-dependent manner [3]. To assess whether this apoptosis was triggered by a virus-dependent induction of cellular stress, we first focused on malfunctions at the level of the ER. We took advantage of a previously described recombinant CDV (rgA75/17-V, which efficiently infects Vero cells and expresses high amount of e-GFP) to monitor infection. Importantly, this virus induces a non-cytolytic, persistent type of infection in many cell types including Vero cells. Indeed, no obvious signs of cyotpathic effects are seen up to 6 days post infection and infected cells can efficiently be passaged several times before cyotpathic effects can be observed [10]. This recombinant virus rgA75/17-V is referred to in this study as CDV to simplify the nomenclature. 24 hours post-infection, we monitored CRT expression as a marker of ER stress [18], [19] by means of immunofluorescence in fixed and permeabilized Vero cells. In GFP positive infected cells, CRT staining was strongly enhanced (Figure 1B, middle panel, white arrow head) as compared to neighbouring non-infected cells or control culture (Figure 1A and B). Immunostaining of two additional ER stress markers -the proapoptotic transcription factor CHOP/GADD 153 [6] and the chaperon protein calnexin [5]- specifically stained infected cells, confirming the CDV-mediated ER stress induction (Figure 1C and D, white arrows heads). To confirm these data in a quantitative manner, increased expression of all markers (CRT, CHOP/GADD 153, and calnexin) in CDV-infected cells was monitored by flow cytometry (Figure 1E). 36 hours post-infection, CDV-infected cells (as sorted by GFP expression) revealed a significant increase of all three ER stress marker expressions. In a sharp contrast, in non-infected cells of the same culture (GFP-), ER stress markers did not significantly differ from isotype control (Figure 1E).

### The viral glycoproteins F and H induce ER stress and alteration in Ca^2+^ homeostasis

**Figure 2:**
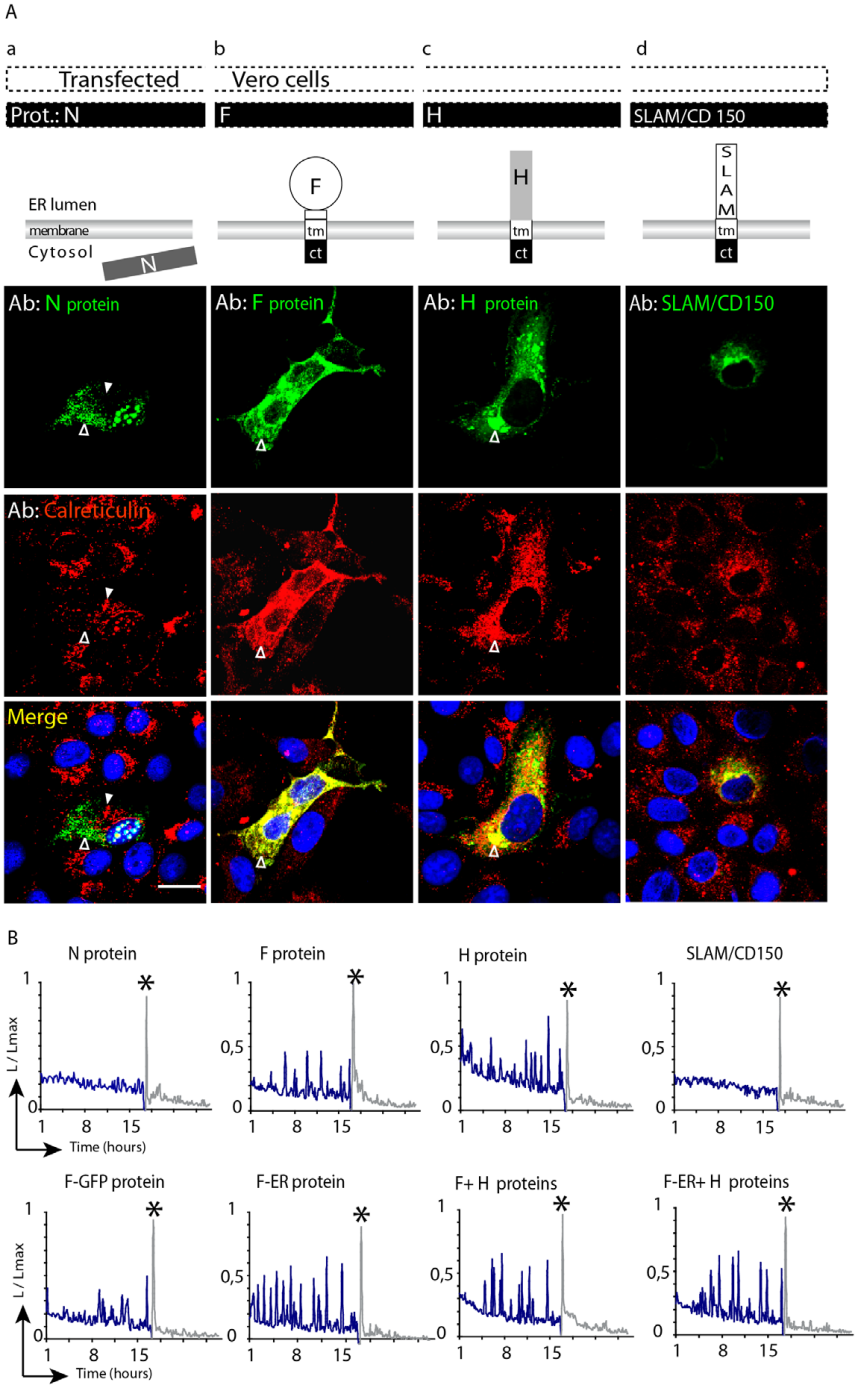
CDV F and H surface glycoproteins accumulate in the ER, induce increased CRT expression and cause changes in Ca^2+^ homeostasis in Vero cells. (**A**) Vero cells were transiently transfected with plasmids expressing CDV protein N (Aa), F (Ab), H (Ac) or the SLAM/CD150 construct (Ad). Top row: schematic representation of their respective protein structures. Top panels: immunofluorescence at 24 hours post-transfection for N, F, H, and SLAM/CD150 (in green). Middle panels: immunofluorescence for CRT (in red). Bottom panels show merged images, including nuclear staining with DAPI (in blue). The viral F and H glycoproteins accumulated within the ER lumen and co-localize with strongly expressed CRT (empty white arrow heads). In contrast, cytosolic N CDV protein is not localized to the ER and does not induce CRT upregulation. (Compare empty white arrow head corresponding to N CDV protein, and white arrow head indicating CRT). SLAM/CD150 does not induce CRT upregulation. Scale bars, 30 µm. The immunofluorescence assays were performed in fixed and permeabilized cells. (**B**) Investigation of Ca^2+^ homeostasis. Vero cells were co-transfected with *GFP*-aequorin (GA) and different combination of the CDV glycoproteins and SLAM/CD150, as indicated above each panel. 24 hours after transfection, 5 mM Coelenterazine was added and photon counting started 60 minutes later. At the end of the experiment 2 mM of ionomycin was added to completely empty ER Ca^2+^ stores, followed by high Ca^2+^ solution (10 mM, shown by the last peak “High/Ca^2+^” and represented by an asterisk (*). This enabled an estimation of GA expression levels and allows normalization between the acquisitions.

We next investigated whether both CDV surface glycoproteins, F and H, were sufficient to induce the aforementioned ER stress. Vero cells were transiently transfected with F or H-expressing plasmids and immunofluorescence in fixed and permeabilized cells was performed 24 hours post-infection (Figure 2Ab and Ac). As expected, the staining indicated that both proteins were localized along the secretory pathway up to the plasma membrane, though cell surface staining in fixed and permeabilized cells remained moderate. However, efficient cell surface expression of both proteins has already been demonstrated [21]–[24]. Importantly, the staining was clearly enhanced around nuclei, suggesting ER accumulation. Indeed, co-staining of CRT, as a widely used marker of ER localisation [20], with F or H clearly indicated their co-localization, confirming the expected F and H targeting into the endoplasmic reticulum compartment (Figure 2Ab–c). Hence, similar to results obtained in CDV-infected cells (Figure 1), Vero cells transiently expressing F and H exhibited accumulation of both glycoproteins in the ER, a phenotype that correlated with CRT over-expression (Figure 2Ab and 2Ac). Conversely, there was no significant CRT upregulation in non-transfected cells (data not shown), in cells expressing the cytosolic CDV nucleocapsid protein (N) or in cells expressing an unrelated glycoprotein (the signaling lymphocytic activation molecule -SLAM/CD150-), (Figure 2Aa and 2Ad). Together, these results strongly support the hypothesis that CDV-induced ER stress is mediated by the accumulation of F and H in the ER compartment.

We previously documented that CDV could induce a significant release of L-glutamate from hippocampal primary cell cultures. Interestingly, it has been suggested that following virus-induced ER-stress, Ca^2+^ may be released from the ER [25]–[27]. We thus investigated whether intracellular Ca^2+^ is released as a result of CDV glycoproteins expression. This was achieved by using the GFP-aequorin reporter assay [16], [17]. Figure 2B illustrates that Ca^2+^ signals in Vero cells transfected with the nucleocapsid gene (N) did not show any differences in the recorded signals as compared to that obtained with the unrelated SLAM/CD150 gene. In contrast, in CDV F-, H- or F/H-expressing cells, substantial increased Ca^2+^ signals were recorded, corresponding to release of Ca^2+^ in the cytosol (Figure 2B, F, H and F+H). We also tested cells which expressed an engineered F protein that bears the GFP fused to its c-terminal domain, allowing for a direct assessment of F localisation in transfected cells without drastically impeding F functionality (not shown). This protein triggered a similar enhancement of Ca^2+^ cytosolic release (Figure 2B, F-GFP). Finally, as a positive control of ER-stress induction, we expressed an F protein which contains an ER-retention tag signal (KDEL) fused to its cytosolic tail (F-ER) and consequently exclusively accumulates in the ER (not shown), as anticipated [21]. F-ER did potently trigger Ca^2+^ cytosolic release when it was expressed alone or co-expressed with H (Figure 2B, F-ER and F-ER+H, respectively).

### Hippocampal cultures transfected with CDV glycoproteins show ER stress and altered Ca^2+^ homeostasis

**Figure 3:**
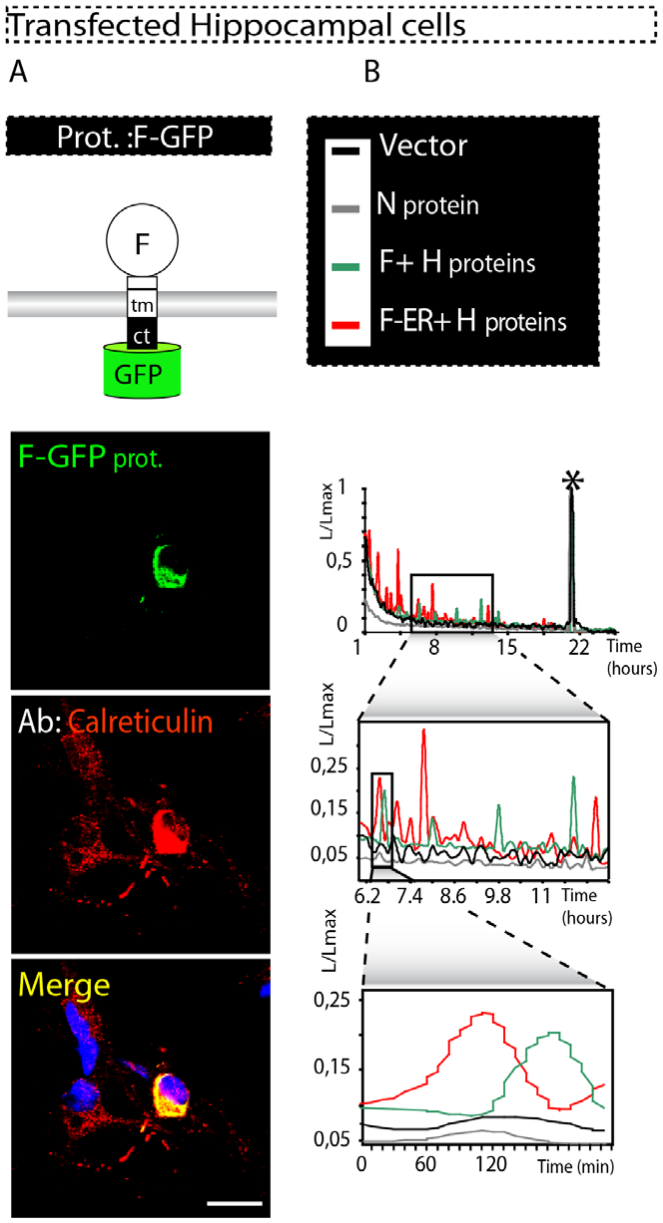
CDV F and H surface glycoproteins accumulate in the ER, induce increased CRT expression and cause changes in Ca^2+^ homeostasis in primary hippocampal cells. (**A**) Top panel: Drawing representing the F-GFP construct. F-GFP was transfected into primary hippocampal neurons. Bottom panels: visualisation of cells expressing F (green fluorescence) and CRT (red fluorescence). For CRT staining, immunofluorescence was performed in fixed and permeabilized cells. The two panels are merged together with DAPI staining in the very bottom panel. As in Vero cells, this chimera induced an increased CRT expression. Scale bars, 30 µm. (**B**) Investigation of Ca^2+^ homeostasis. Primary hippocampal neurons were co-transfected with empty vector and the CDV protein-expressing vectors as indicated in the top panel. Below, the graphs exhibit combined curves of Ca^2+^ release in the cytosol upon expression of the indicated proteins. The bottom graph is a magnification of the middle graph, which is a magnification of the top graph. All samples were analysed in triplicate on three separate experiments, and one representative experiment is shown here. The recorded Ca^2+^ responses are consistent with the ER F and H protein accumulation and subsequent ER stress observed in Vero cells.

To investigate whether the ER stress was also induced by CDV in a more physiologically relevant model, dissociated cells derived from rat brain hippocampus were used [12]. Consistent with results obtained in Vero cells, Figure 3A indicates that F-GFP (most detection-sensitive mutant) strongly accumulated in the ER. As we previously observed in Vero cells, accumulation in the ER of F-GFP correlated with significant enhancement of CRT expression, in turn reflecting ER stress induction. Similar results were observed with untagged F or H proteins (not shown).

We next assessed the alteration of Ca^2+^ homeostasis in primary rat brain cells. As seen in Figure 3B, cells transfected with empty vector or N-expressing plasmid did not reveal any enhanced cytoplasmic Ca^2+^ release, whereas co-expression of F and H glycoproteins resulted in a marked increase in cytosolic Ca^2+^ concentration. These data were further validated by our ER-stress triggering control (F-ER) that was here co-expressed with H, and which resulted in even stronger Ca^2+^ release than in F and H-expressing cells (Figure 3B). Taken together, our results obtained in Vero cells were confirmed in primary rat brain cells. Indeed, expressing both CDV glycoproteins in primary neuronal cells led to ER-stress induction and ER calcium-depletion.

### CRT fragmentation is specifically induced by CDV infection

**Figure 4:**
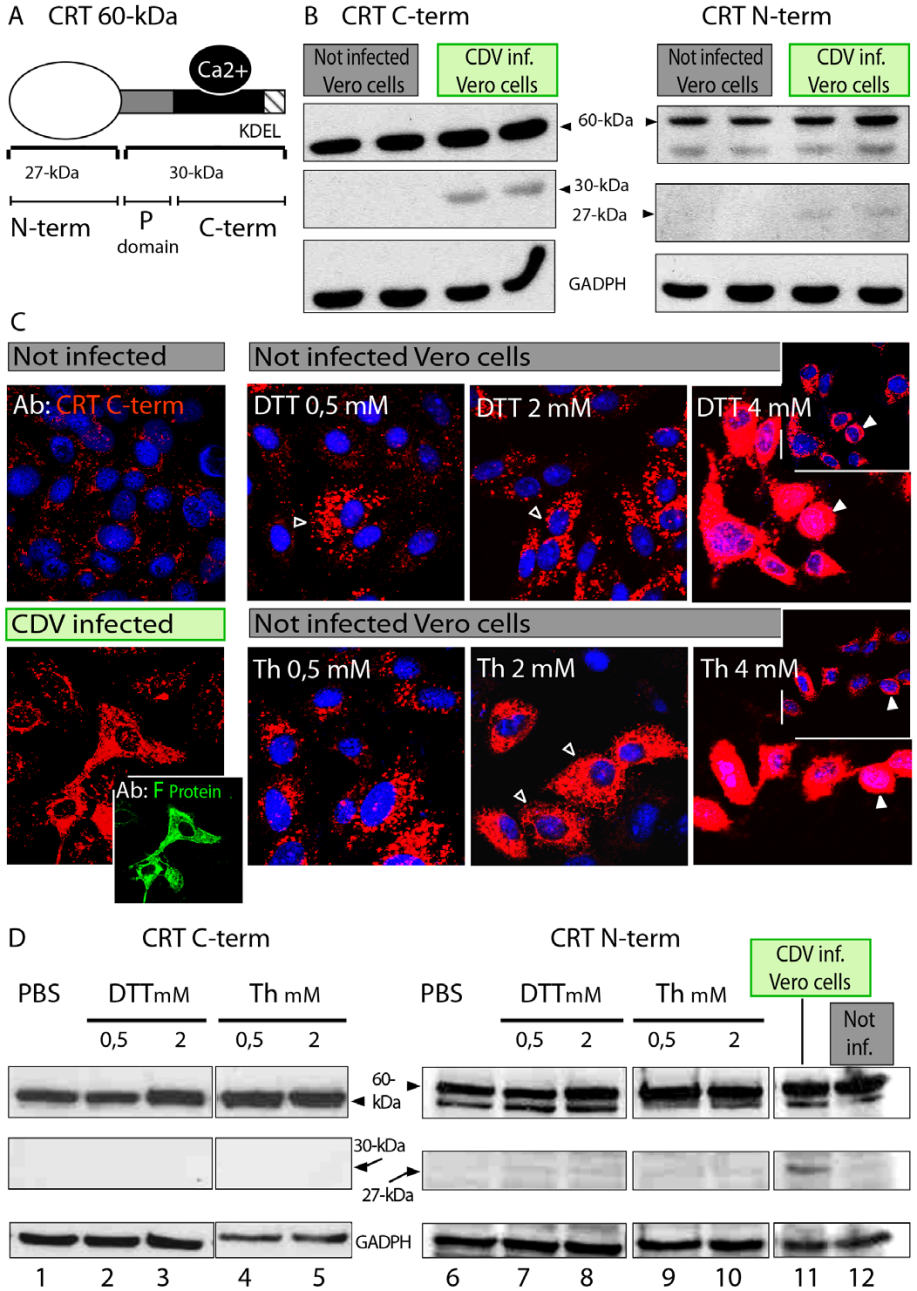
CDV infection of Vero cells causes CRT fragmentation with vasostatin formation. (**A**) Schematic representation of the 60 kDa CRT protein. The globular 27 kDa N-terminal domain (N-term) is the most important antigenic site of the protein. This domain, as well as the P domain, possesses the chaperon function. C-terminal domain (C-term) is important for Ca^2+^ storage and possesses the KDEL ER retention signal. P and C-terminal domains have together an estimated mass of 30 kDa. (**B**) The Vero cells were either left non-infected or infected with CDV. At 48 h post-infection cells were lysed and analyzed by Western blot using C-terminal-specific (left) or N-terminal-specific (right) antibodies. Note the C-terminal 30 kDa fragment, and the 27 kDa N-terminal fragment. GAPDH is used as an internal control. (**C**) Impact of the ER stress inducing drugs dithiothreitol (DTT) and thapsigargin (Th) on CRT expression during CDV infection. Red fluorescence in all panels corresponds to CRT immunostaining, which increases in a DTT concentration-dependent manner (top panels) or in a thapsigargin concentration-dependent manner (white arrow heads). For comparison, calreticulin staining is shown in infected cells probed by immunostaining of the F protein (bottom left and insert panels). Immunofluorescence analyses were performed in fixed and permeabilized cells. (**D**) Western blot using the antibody recognizing either the N- terminal domain of CRT (27 kDa, right panel) or the C- terminal domain (30 kDa; left panel). Both antibodies recognize the full-length CRT (top line, 60 kDa). Cellular extracts come from Vero cells exposed to DTT or thapsigargin (Th), or CDV infected as indicated. GAPDH was used as an internal control. CRT cleavage was specifically mediated by CDV infection (line 11) and not by exposure to DTT or thapsigargin.

CRT is not only used as a marker of ER stress induction (when upregulated) but is also an important contributor to Ca^2+^ homeostasis. We thus explored whether the structure and/or localization was altered in CDV-infected cells. In a first series of experiments, Western blots were performed in order to analyze CRT during infection. Two different CRT antibodies were used: a monoclonal antibody (employed in the previous staining experiments) that recognizes the C-terminal part of the protein, and a polyclonal antiserum, which is directed against the N-terminal part of the protein (Figure 4A).

While the full-length CRT protein was detected to similar extend in infected and non-infected cells, an additional fragment of about 30 kDa was exclusively observed in CDV-infected cells. This 30 kDa band was recognized by the C-terminal-specific antibody (Figure 4B, left blot). The apparent low levels of cleaved fragments correspond to the fact that only 15% of the cells were infected at the time of cell lysis, as evaluated by counting GFP positive cells. We sought to detect the second putative cleavage product of the full-length CRT, *i.e.* the N-terminal region using the N-terminal CRT antiserum. We detected the full-length CRT protein and a fragment of about 27 kDa, which corresponded to the N-terminal CRT fragment called vasostatin [28], in addition to several smaller bands (not shown) (Figure 4B, right blot).

To investigate whether CRT fragmentation is specifically dependent on CDV infection, we assessed the effect of two drugs: dithiothreitol (DTT) and thapsigargin (Th). Both drugs are known to induce ER stress by two different mechanisms: inducing protein misfolding or blocking ER Ca^2+^ ATPase pump activity, respectively [29]. In DTT- and Th-treated Vero cells, there was a clear dose-dependent accumulation of CRT in the ER, compared to non-treated cells, as revealed by immunofluorescence analysis of fixed and permeabilized cells (Figure 4C). The highest drug concentration (4 µM) exhibited the most severe CRT increase. Higher drug concentrations were clearly cytotoxic (data not shown). Strikingly, neither DTT nor Th treatment induced detectable CRT fragmentation (immunoblot Figure 4D lanes 1–5 and 6–10), whereas in CDV-infected cells, a clear band migrating with a molecular weight of about 27 kDa was present (right immunoblot, lane 11). In summary, our cellular and biochemical data provide strong evidence that CRT fragmentation is specifically mediated by CDV infection. Because drug-induced cellular ER stress did not cause any detectable increase of the CRT cleavage process, our results suggest that virus-induced ER stress may be different in some way from that induced by DTT or Th. Alternatively, CRT cleavage in CDV-infected cells might require additional effects that viral glycoprotein expression would induce.

### CDV-dependent re-localisation of the 27 kDa vasostatin CRT fragment at the plasma membrane

**Figure 5:**
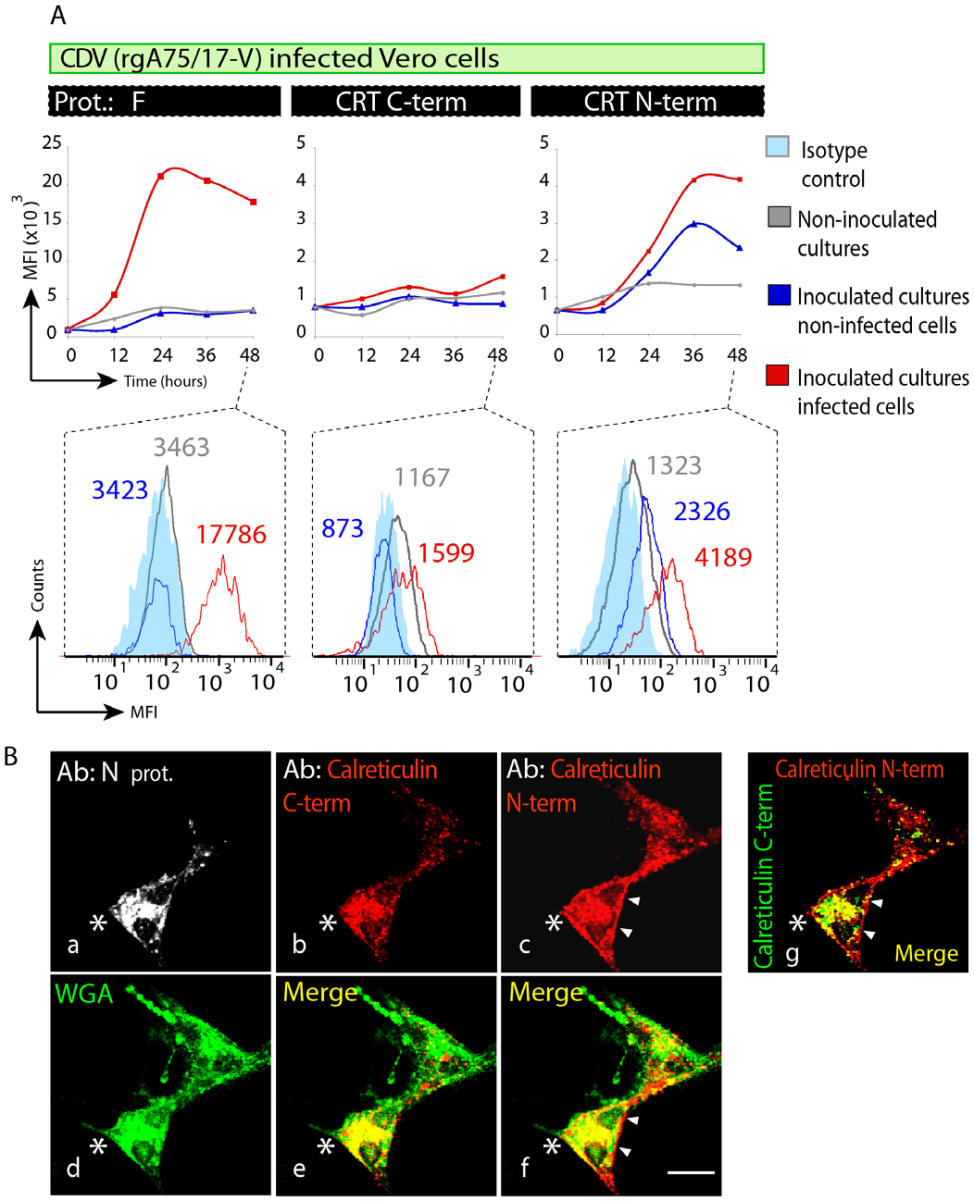
CRT N-terminal fragments are re-localized at the cell surface. (**A**) The kinetics of the appearance of the F protein and CRT C-terminal and N-terminal fragments at the surface of infected Vero cells were monitored by flow cytofluorimetry using the corresponding specific antibodies. Control cell cultures were not infected (grey lines). From infected cell cultures, infected cells (red line; GFP-positive) and non-infected cells (blue line, GFP-negative) were identified by virus-encoded GFP fluorescence. Cell surface immuno-labelling of cells (unifxed and nonpermeablized) with F and CRT C-terminal and N-terminal were performed with specific antibodies as indicated at the top of the panels. The mean fluorescence intensity (MFI) of the labelling was determined within each cell population regularly over 48 hours of infection (top panels). The three bottom panels represent the distribution of the MFI within each population at 48 hours post-infection. Here is shown one representative experiment out of three independent experiments. (**B**) Membrane localisation of CRT N-terminal fragment following CDV infection. At 24 hours post-infection in Vero cells, cultures were immuno-labelled for the viral F protein to identify infected cells (Ba; fluorochrome: FITC), C-terminal specific anti-CRT antibody (Bb; fluorochrome: CY3), and N-terminal specific anti-CRT antibody (Bc; fluorochrome: CY5). In panel Bd, cell membranes were stained with alexa-405-conjugated wheat germ agglutinin (WGA). The merges images b and d (Be) reveal little co-localisation of CRT C-terminal fragment with the cell surface, while the merges images c and d (Bf) indicate partial surface localization of the CRT N-terminal fragment. Panel g is a merge between images b and c. Scale bar, 30 µm. Immunofluorescence analyses were performed in fixed and permeabilized cells.

Cleavage of CRT is known to release the N-terminal fragments, also known as vasostatin [28]. To determine if the vasostatin is released from infected cells, Vero cells were infected with CDV for 24 hours, trypsinized and re-plated into new wells for additional time periods. This was performed to ascertain synchronization of the putative release of CRT fragments between all infected wells. To ascertain specific cell surface-exposed CRT staining, immunofluorescence was performed on non-fixed and non-permeabilized cells at 4°C to prevent internalisation. Quantitative data were recorded by cytofluorimetry. Infected cells could be distinguished from non-infected cells by their GFP expression from the recombinant virus. As a control for surface protein expression, we used an anti-F antiserum, which demonstrated an increase in mean fluorescence intensity (MFI) by 12 hours after re-plating, and this, only in infected cells (Figure 5A; F). We saw no significant enhanced signals from infected cells using the antibody targeting the CRT C-terminus (Figure 5A; CRT C-term). In contrast, a substantial increase was recorded when using the N-terminal antiserum in both the CDV-inoculated cultures and the non-infected cells, suggesting secretion of vasostatin and binding to cell surface receptor (Figure 5A; CRT N-term). Non-infected cultures did not stain with this antibody, indicating that it was specifically due to the presence of infected cells. We then investigated by means of immunofluorescence the sub-cellular localisation of CRT fragments in CDV-infected cells. This was performed in fixed and permeabilized cells. While CRT staining remained almost exclusively cytosolic using the C-terminal-recognizing antibody, the N-terminal-recognizing antibody illuminated both the cytosol and the plasma membrane. Furthermore, co-staining of CRT with WGA, a marker for all cell membranes, revealed that only the N-terminal fragment of the CRT co-localized with the marker (Figure 5Bf).

In summary, we demonstrated that both CRT antibodies stained the cytosol but that only the N-terminal-recognizing antibody stained the plasma membrane. This specific recognition of the N terminal fragment at the cell surface was confirmed by cytofluorimetry and immunofluorescence assays. Altogether, this provides strong evidence that only N-terminal fragments of the CRT, but not the full-length protein or C-terminal fragments, are re-localized to the plasma membrane of the infected and neighbouring non-infected cells. This pattern is consistent with the N-terminal fragment being the vasostatin, a secreted, biologically active fragment of CRT.

## Discussion

In this study we explored the molecular events associated with ER stress induced by *Morbillivirus* infection and transient expression of viral glycoproteins. CRT is a luminal ER chaperon implicated in the folding of newly synthesized proteins, a component of the ER quality control system [9], [30], [31]. The N-terminal and the central P domains of CRT display the chaperon function and can bind the hydrophobic parts of the nascent proteins in the ER thus preventing protein aggregation [32]. In addition, by accumulating Ca^2+^ in its C-terminal domain [14], [33], CRT is the major Ca^2+^-binding and buffering protein in the ER lumen [34], [35]. During virus infection, viral glycoproteins are folded and glycosylated in the ER and further processed in the Golgi apparatus. While the release of Ca^2+^ from ER stores appears to be the primary initiator of the ER stress response and cellular apoptosis [25]–[27], [36]–[38], the primary mechanism responsible for the disruption of calcium homeostasis remains unknown. Herein we report for the first time that accumulation of CDV glycoproteins in the ER, CRT over-expression and ER stress correlated very well with the disruption of ER Ca^2+^ homeostasis of infected cells. Importantly, our results obtained in Vero cells were recapitulated in primary rat hippocampal culture. Furthermore, we showed that CDV could induce CRT fragmentation and selective secretion of the CRT N-terminal fragment, also known as vasostatin, and its binding to cell surface to both infected cells and neighbouring non-infected cells.

Depletion of Ca^2+^ from ER stores, an event potentially triggered by ER stress, critically affects the survival of CNS cells by inducing proapoptotic stimuli and the exocytotic release of synaptic vesicles [39]–[41]. These events could explain the apoptotic death of CNS-infected cells [42] as well as apoptosis of neighbouring non-infected brain cells as a result of Ca^2+^-induced L-glutamate release [3]. In the CNS, both neurons and glial cells can be infected by CDV in dogs and other carnivores [1]. In the early stage of infection, CDV causes an acute infection followed by a subacute stage leading in some cases to chronic infection. During acute infection, demyelination has been described as being a direct consequence of virus replication, in the absence of detectable inflammation [1]. In contrast, during chronic demyelination, plaque progression seems to be mainly related to an immunopathological process [43]. Based on these and our past [3] and present observations, we suggest that in the acute phase of the infection in the CNS, neurodegenerative events are at least in part initiated by an ER stress, which increases CRT expression and perturbs Ca^2+^ homeostasis, subsequently releasing L-glutamate [3]. Altered Ca^2+^ homeostasis and L-glutamate release in turn cause rapid CNS degeneration (within days or a week) corresponding to the acute phase of the disease. The fact that we observed this cascade of molecular events in the immune deficient Vero cells does not exclude that *in vivo* some more primordial defence system of the infected cells contribute to the initiation of the neurodegenerescence.

**Figure 6:**
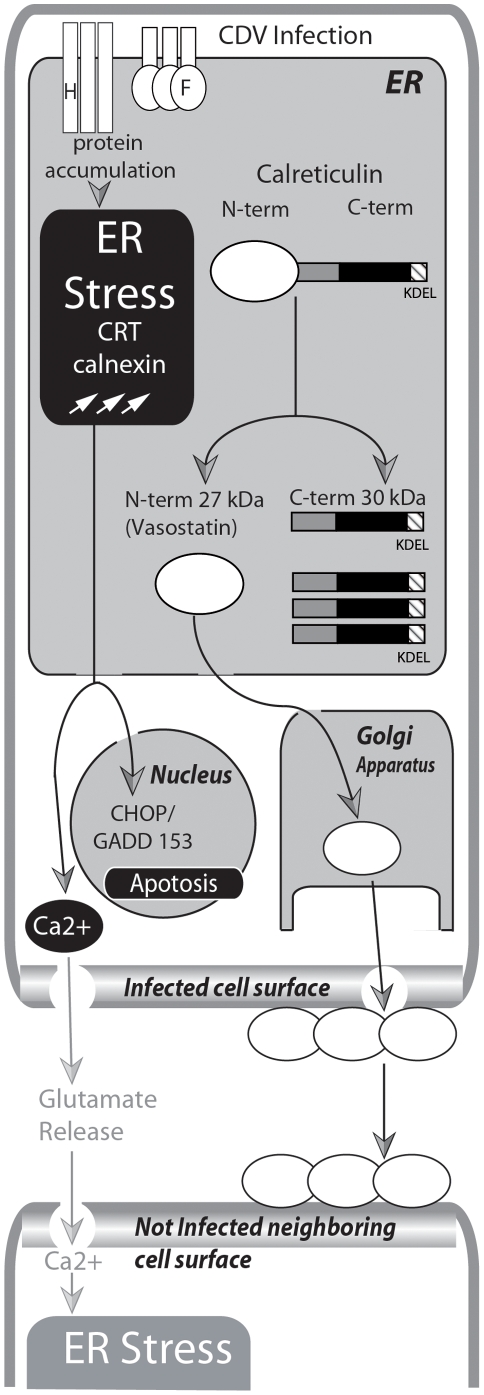
Mechanistic model of neurodegenerative processes induced by CDV infection. The F and H CDV proteins are accumulating in the ER. This event induces an early ER stress event. In early ER stress, the quantities of CRT chaperon increase, the Ca^2+^ homeostasis is altered and Ca^2+^ is depleted from ER stores. Increase of cytosolic Ca^2+^ can have as consequence a glutamate release during CDV infection as previously described [3]. Glutamate release could induce, in the neighbouring neurons, Ca^2+^ entry followed by an ER stress induction [56]. During ER stress, the infected cells show enhance expression of the chaperons CRT, calnexin and GRP94 and relocalisation of the transcription factor ATF-6 in the nucleus followed by the expression of the proapoptotic factor CHOP/GADD 153. More importantly, infected cells show CRT fragmentation in a CDV-dependent manner. C-terminal fragments are retained in the ER by the KDEL signal whereas CRT N-terminal fragments are present after 24 hours at the cell surface. Cell surface exposition of CRT N-terminal fragment may contribute to CDV-mediated neurodegenerative auto-immunity. In ***grey italic*** are events described in previous publications.

Under physiological conditions, full-length CRT [35] or N-terminal fragments [44] have been detected in small amounts at the cell surface. Importantly, the adaptive immune system is strongly activated by increasing amounts of full-length CRT [45], [46] or by vasostatin, the 27 kDa N-terminal CRT fragment [28] at the cell surface. Here, we reported that CDV can also trigger CRT fragmentation and relocation of the N-terminal vasostatin CRT fragment to the cell surface of both infected and non-infected cells (Figure 6). Though CRT cleavage appeared to be caused by CDV-induced ER stress, the fact that the well-known ER stress inducers thapsigargin and DTT do not cause CRT cleavage, suggest that the triggering of ER stress may not be equivalent in both conditions. Interestingly, cell surface localization or secretion of the vasostatin has been first described in Epstein-Barr-Virus (EBV)-infected cells [44], [47] although the precise molecular mechanism underlying this phenomenon remains undetermined. Pike and co-workers additionally demonstrated that vasostatin efficiently inhibited endothelial cell proliferation and could supressed angiogenesis *in vivo* [28]. In addition, vasostatin also significantly reduced tumor growth in mice [47]. While one possible explanation to explain CDV-induced CRT cleavage would implicate a direct role of F and/or H on CRT processing and subsequent secretion of the vasostatin from the cell, any other indirect effects cannot be rule out. Further works are hence warranted to illuminate the precise molecular mechanism leading to CRT cleavage in CDV-infected cells. Moreover, we speculate that secretion of the CDV-induced N-terminal vasostatin fragment may contribute to the establishment of the chronic phase of the disease over many months or years. Indeed, vasostatin may stimulate the adaptive immune system, which may generate auto-antibodies directed against it. This alteration might even be amplified by the fact that we detected the vasostatin on the surface of non-infected cells in infected cultures, suggestive of a molecular mechanism by which the released CRT fragments might bind to an unknown receptor on the surface of neighbouring cells [44], [46] (Figure 6). Increased CRT-specific auto-antibodies have been reported in autoimmune diseases such as Sjögrens's syndrome and systemic lupus erythematosus. In some cases these diseases show neurodegenerative progression [44], [48], [49]. It is interesting to note that early stages of multiple sclerosis and systemic lupus erythematosus share some clinical signs than those induced by CDV [50]. Alternatively, vasostatin-dependent inhibition of angiogenesis in the brain may also contribute to CDV-induced neurological disorders.

Additional effects of CRT cell surface exposure are known in cancer progression and therapies. Indeed, in mouse models, exposure of CRT on a tumour cell surface following treatment with chemotherapeutic agents caused apoptosis, tumour immunogenicity and cancer cell death [45]. Consistent with these finding, CDV and MV infections showed promising oncolytic activity in a mouse model of lymphoma carcinomas [51]. The MV-mediated anti-tumour response has been characterized as an immune response triggering cell apoptosis in hepatocellular carcinoma and human cutaneous T cell lymphoma [52]–[54]. The fact that MV infection in human lymphoma induces ER-resident stress proteins [55] is in line with our own observations linking viral infection-mediated ER stress and CRT exposed at the cell surface. While the molecular mechanisms of oncolytic action mediated by CDV and MV very likely are governed by F/H-induced syncytium formation and subsequent cell lysis, they are not yet completely elucidated. Thus, it is tempting to speculate that vasostatin on the cell surface of both infected and neighbouring cells may contribute to anti-tumour activity *in vivo*.

In conclusion, we demonstrate that during morbillivirus infection the accumulation of viral surface glycoproteins in the ER induces an ER stress characterized by Ca^2+^ release, apoptosis and CRT fragmentation. The N-terminal fragment of CRT, vasostatin, is secreted and binds to the cell surface of both infected cells and neighbouring cells. An auto-antibodies response to vasostatin, as described in other disease, might result in neurodegenerative-autoimmunity.

## References

[1] Vandevelde M, Zurbriggen A (2005). Demyelination in canine distemper virus infection: a review.. Acta Neuropathol (Berl).

[2] Beineke A, Puff C, Seehusen F, Baumgartner W (2009). Pathogenesis and immunopathology of systemic and nervous canine distemper.. Vet Immunol Immunopathol.

[3] Brunner JM, Plattet P, Majcherczyk P, Zurbriggen A, Wittek R (2007). Canine distemper virus infection of primary hippocampal cells induces increase in extracellular glutamate and neurodegeneration.. J Neurochem.

[4] Kaufman RJ (1999). Stress signaling from the lumen of the endoplasmic reticulum: coordination of gene transcriptional and translational controls.. Genes Dev.

[5] Malhotra JD, Kaufman RJ (2007). The endoplasmic reticulum and the unfolded protein response.. Semin Cell Dev Biol.

[6] Szegezdi E, Logue SE, Gorman AM, Samali A (2006). Mediators of endoplasmic reticulum stress-induced apoptosis.. EMBO Rep.

[7] Marciniak SJ, Yun CY, Oyadomari S, Novoa I, Zhang Y (2004). CHOP induces death by promoting protein synthesis and oxidation in the stressed endoplasmic reticulum.. Genes Dev.

[8] Oyadomari S, Mori M (2004). Roles of CHOP/GADD153 in endoplasmic reticulum stress.. Cell Death Differ.

[9] Michalak M, Corbett EF, Mesaeli N, Nakamura K, Opas M (1999). Calreticulin: one protein, one gene, many functions.. Biochem J.

[10] Plattet P, Zweifel C, Wiederkehr C, Belloy L, Cherpillod P (2004). Recovery of a persistent Canine distemper virus expressing the enhanced green fluorescent protein from cloned cDNA.. Virus Res.

[11] Cherpillod P, Beck K, Zurbriggen A, Wittek R (1999). Sequence analysis and expression of the attachment and fusion proteins of canine distemper virus wild-type strain A75/17.. J Virol.

[12] Steiner P, Sarria JC, Glauser L, Magnin S, Catsicas S (2002). Modulation of receptor cycling by neuron-enriched endosomal protein of 21 kD.. J Cell Biol.

[13] Hamburger D, Griot C, Zurbriggen A, Orvell C, Vandevelde M (1991). Loss of virulence of canine distemper virus is associated with a structural change recognized by a monoclonal antibody.. Experientia.

[14] Corbett EF, Michalak KM, Oikawa K, Johnson S, Campbell ID (2000). The conformation of calreticulin is influenced by the endoplasmic reticulum luminal environment.. J Biol Chem.

[15] Shimomura O (1997). Membrane permeability of coelenterazine analogues measured with fish eggs.. Biochem J.

[16] Curie T, Rogers KL, Colasante C, Brulet P (2007). Red-shifted aequorin-based bioluminescent reporters for in vivo imaging of Ca2 signaling.. Mol Imaging.

[17] Rogers KL, Stinnakre J, Agulhon C, Jublot D, Shorte SL (2005). Visualization of local Ca2+ dynamics with genetically encoded bioluminescent reporters.. Eur J Neurosci.

[18] Vecchi C, Montosi G, Zhang K, Lamberti I, Duncan SA (2009). ER stress controls iron metabolism through induction of hepcidin.. Science.

[19] Nguyen TO, Capra JD, Sontheimer RD (1996). Calreticulin is transcriptionally upregulated by heat shock, calcium and heavy metals.. Mol Immunol.

[20] Mertens E, Kajaste-Rudnitski A, Torres S, Funk A, Frenkiel MP (2010). Viral determinants in the NS3 helicase and 2K peptide that promote West Nile virus resistance to antiviral action of 2′,5′-oligoadenylate synthetase 1b.. Virology.

[21] Plattet P, Cherpillod P, Wiener D, Zipperle L, Vandevelde M (2007). Signal peptide and helical bundle domains of virulent canine distemper virus fusion protein restrict fusogenicity.. J Virol.

[22] Plattet P, Langedijk JP, Zipperle L, Vandevelde M, Orvell C (2009). Conserved leucine residue in the head region of morbillivirus fusion protein regulates the large conformational change during fusion activity.. Biochemistry.

[23] Wiener D, Plattet P, Cherpillod P, Zipperle L, Doherr MG (2007). Synergistic inhibition in cell-cell fusion mediated by the matrix and nucleocapsid protein of canine distemper virus.. Virus Res.

[24] Zipperle L, Langedijk JP, Orvell C, Vandevelde M, Zurbriggen A (2010). Identification of key residues in virulent canine distemper virus hemagglutinin that control CD150/SLAM-binding activity.. J Virol.

[25] Aldabe R, Irurzun A, Carrasco L (1997). Poliovirus protein 2BC increases cytosolic free calcium concentrations.. J Virol.

[26] Ali-Furet NL, Chami M, Houel L, De GF, Vernejoul F (2005). Hepatitis C virus core triggers apoptosis in liver cells by inducing ER stress and ER calcium depletion.. Oncogene.

[27] Deniaud A, Sharaf eldO, Maillier E, Poncet D, Kroemer G (2008). Endoplasmic reticulum stress induces calcium-dependent permeability transition, mitochondrial outer membrane permeabilization and apoptosis.. Oncogene.

[28] Pike SE, Yao L, Jones KD, Cherney B, Appella E (1998). Vasostatin, a calreticulin fragment, inhibits angiogenesis and suppresses tumor growth.. J Exp Med.

[29] Shen J, Prywes R (2005). ER stress signaling by regulated proteolysis of ATF6.. Methods.

[30] Krause KH, Michalak M (1997). Calreticulin.. Cell.

[31] Nakamura K, Zuppini A, Arnaudeau S, Lynch J, Ahsan I (2001). Functional specialization of calreticulin domains.. J Cell Biol.

[32] Saito Y, Ihara Y, Leach MR, Cohen-Doyle MF, Williams DB (1999). Calreticulin functions in vitro as a molecular chaperone for both glycosylated and non-glycosylated proteins.. EMBO J.

[33] Ellgaard L, Riek R, Herrmann T, Guntert P, Braun D (2001). NMR structure of the calreticulin P-domain.. Proc Natl Acad Sci U S A.

[34] Gelebart P, Opas M, Michalak M (2005). Calreticulin, a Ca2+-binding chaperone of the endoplasmic reticulum.. Int J Biochem Cell Biol.

[35] Johnson S, Michalak M, Opas M, Eggleton P (2001). The ins and outs of calreticulin: from the ER lumen to the extracellular space.. Trends Cell Biol.

[36] Hetz C, Russelakis-Carneiro M, Maundrell K, Castilla J, Soto C (2003). Caspase-12 and endoplasmic reticulum stress mediate neurotoxicity of pathological prion protein.. EMBO J.

[37] Rutkowski DT, Kaufman RJ (2004). A trip to the ER: coping with stress.. Trends Cell Biol.

[38] Voccoli V, Mazzoni F, Garcia-Gil M, Colombaioni L (2007). Serum-withdrawal-dependent apoptosis of hippocampal neuroblasts involves Ca++ release by endoplasmic reticulum and caspase-12 activation.. Brain Res.

[39] Berridge MJ, Bootman MD, Roderick HL (2003). Calcium signalling: dynamics, homeostasis and remodelling.. Nat Rev Mol Cell Biol.

[40] Parpura V, Haydon PG (2000). Physiological astrocytic calcium levels stimulate glutamate release to modulate adjacent neurons.. Proc Natl Acad Sci U S A.

[41] Pasti L, Zonta M, Pozzan T, Vicini S, Carmignoto G (2001). Cytosolic calcium oscillations in astrocytes may regulate exocytotic release of glutamate.. J Neurosci.

[42] Paschen W (2003). Endoplasmic reticulum: a primary target in various acute disorders and degenerative diseases of the brain.. Cell Calcium.

[43] Summers BA, Appel MJ (1994). Aspects of canine distemper virus and measles virus encephalomyelitis.. Neuropathol Appl Neurobiol.

[44] Eggleton P, Llewellyn DH (1999). Pathophysiological roles of calreticulin in autoimmune disease.. Scand J Immunol.

[45] Obeid M, Tesniere A, Ghiringhelli F, Fimia GM, Apetoh L (2007). Calreticulin exposure dictates the immunogenicity of cancer cell death.. Nat Med.

[46] Gardai SJ, McPhillips KA, Frasch SC, Janssen WJ, Starefeldt A (2005). Cell-surface calreticulin initiates clearance of viable or apoptotic cells through trans-activation of LRP on the phagocyte.. Cell.

[47] Pike SE, Yao L, Setsuda J, Jones KD, Cherney B (1999). Calreticulin and calreticulin fragments are endothelial cell inhibitors that suppress tumor growth.. Blood.

[48] Boehm J, Orth T, Van NP, Soling HD (1994). Systemic lupus erythematosus is associated with increased auto-antibody titers against calreticulin and grp94, but calreticulin is not the Ro/SS-A antigen.. Eur J Clin Invest.

[49] McCauliffe DP, Zappi E, Lieu TS, Michalak M, Sontheimer RD (1990). A human Ro/SS-A autoantigen is the homologue of calreticulin and is highly homologous with onchocercal RAL-1 antigen and an aplysia “memory molecule”.. J Clin Invest.

[50] Ferreira S, D'Cruz DP, Hughes GR (2005). Multiple sclerosis, neuropsychiatric lupus and antiphospholipid syndrome: where do we stand?. Rheumatology (Oxford).

[51] Suter SE, Chein MB, von M, V, Yip B, Cattaneo R (2005). In vitro canine distemper virus infection of canine lymphoid cells: a prelude to oncolytic therapy for lymphoma.. Clin Cancer Res.

[52] Blechacz B, Splinter PL, Greiner S, Myers R, Peng KW (2006). Engineered measles virus as a novel oncolytic viral therapy system for hepatocellular carcinoma.. Hepatology.

[53] Heinzerling L, Kunzi V, Oberholzer PA, Kundig T, Naim H (2005). Oncolytic measles virus in cutaneous T-cell lymphomas mounts antitumor immune responses in vivo and targets interferon-resistant tumor cells.. Blood.

[54] Kunzi V, Oberholzer PA, Heinzerling L, Dummer R, Naim HY (2006). Recombinant measles virus induces cytolysis of cutaneous T-cell lymphoma in vitro and in vivo.. J Invest Dermatol.

[55] Grote D, Russell SJ, Cornu TI, Cattaneo R, Vile R (2001). Live attenuated measles virus induces regression of human lymphoma xenografts in immunodeficient mice.. Blood.

[56] Chen X, Kintner DB, Luo J, Baba A, Matsuda T (2008). Endoplasmic reticulum Ca2+ dysregulation and endoplasmic reticulum stress following in vitro neuronal ischemia: role of Na+-K+-Cl- cotransporter.. J Neurochem.

